# The Relationships between Park Quality, Park Usage, and Levels of Physical Activity in Low-Income, African American Neighborhoods

**DOI:** 10.3390/ijerph16010085

**Published:** 2018-12-30

**Authors:** Megan Knapp, Jeanette Gustat, Revonda Darensbourg, Leann Myers, Carolyn Johnson

**Affiliations:** 1 Department of Global Community Health and Behavioral Sciences, Tulane University, New Orleans, LA 70112, USA; rdarens@tulane.edu (R.D.); cjohnso5@tulane.edu (C.J.); 2 Department of Epidemiology, Tulane University, New Orleans, LA 70112, USA; gustat@tulane.edu; 3 Department of Global Biostatistics and Data Science, Tulane University, New Orleans, LA 70112, USA; myersl@tulane.edu

**Keywords:** parks, neighborhoods, physical activity, African American, built environment, disorder

## Abstract

Parks can be an important, low-cost neighborhood resource to increase physical activity and reduce overweight and obesity. The quality of parks, however, may impact use. This study used observational data to examine the relationships between park quality, park usage and levels of physical activity among users in 31 parks within low-income, African American neighborhoods. Relationships between park use and park characteristics (signs of disorder, attractiveness, and number of activity settings) varied by gender and user activity level. No variables of interest were significant for overall number of male users; whereas, disorder and attractiveness were significant for overall number of female users. Parks with signs of disorder were associated with 49% fewer female users (IRR = 0.51, 95% CI = (0.34–0.77)) and attractive parks with 146% more female users (IRR = 2.46, 95% CI = (1.39–4.33)). Similar significant relationships were found among active but not sedentary female users. Communities may consider increasing park maintenance and addressing attractiveness in existing parks as a relatively low-cost environmental strategy to encourage park use, increase physical activity, and reduce the burden of obesity, especially among women in low-income, African-American communities.

## 1. Introduction

Physical activity offers many health benefits and plays an important role in weight control and prevention of chronic disease [1]. In the United States, only 51.7 percent of adults meet the recommendation of 150 min or more of aerobic physical activity per week [2]. Ecological models suggest that neighborhood environments and characteristics can play a role in an individual’s decision to engage in physical activity [3,4,5].

Research focused on neighborhood resources as determinants of physical activity has found that individuals in low-income, minority neighborhoods have lower levels of physical activity and higher prevalence of overweight and obesity, even after controlling for socioeconomic status [6]. Decreased physical activity in low-income, minority neighborhoods may be partially the result of negative environmental cues, such as vandalism, disrepair, and crime [7,8,9,10]. Following the theory of neighborhood disorder, neighborhoods with high levels of physical disorder (litter, abandoned buildings, graffiti, broken glass) may be less conducive to physical activity [11]. Physical signs of disorder can lead to increased fear of crime, perceived lack of safety, and signal a breakdown of social control [12,13,14,15]. Several studies have indicated that disorder, perceived lack of safety, and fear of crime are barriers to physical activity [14,16,17,18,19].

This same theory may be applied to parks and park usage. Within neighborhoods, parks can be an important, low-cost resource that can provide psychological, social, and physical health benefits for users, and can facilitate community building [4,20,21,22,23]. Park settings offer opportunities for active forms of leisure and can promote neighborhood-based physical activity [4,24,25,26,27,28,29,30]. In one study, parks were identified as the most common place for physical activity among residents in the study neighborhoods [30]. Parks can be a positive community asset and encourage healthy behaviors among residents; however, when they are not maintained, have signs of disorder, and/or are unattractive, residents may have concerns about safety, which may impact use [31,32]. Studies that have examined the quality of specific park assets, incivilities, and conditions of activity setting areas have found positive associations between the quality of park characteristics and the number of users and their level of park-based physical activity [27,33,34,35]. In one study, perceptions of conditions of recreational facilities, such as pleasantness and tidiness, were found to be more strongly associated with park-based physical activity behaviors and weight than availability of and access to the facilities [24].

Studies have shown that low-income, minority neighborhoods have access to parks, but the parks in these neighborhoods are often in poorer condition than those in higher-income, non-minority neighborhoods [19,36,37,38,39,40]. As conditions and perceptions of parks may be more important than the presence or absence of these resources in influencing physical activity, improving quality of the parks may be key to increasing usage and promoting physical activity within these neighborhoods.

While some studies have examined specific park features and used neighborhood-level or self-report data to draw conclusions, few observational studies have examined the relationship between overall park conditions, park usage, and physical activity levels, especially within low-income, minority neighborhoods. To fill this gap and build upon the evidence, this study used observational data to explore the relationships between park disorder, attractiveness, activity settings, park usage, and levels of physical activity in low-income, African American neighborhoods in New Orleans, Louisiana. We hypothesized that attractive parks with less disorder and more activity settings will have a greater number of users and users participating in more physical activity. Identifying specific park characteristics and conditions that influence park-based physical activity can be important to guide park improvements, policy, and programming, and to inform environmental interventions to promote physical activity and reduce sedentary behavior.

## 2. Materials and Methods

### 2.1. Design

Data for this study were drawn from cross-sectional, observational data collected in 31 New Orleans parks. The primary outcome of interest was number of park users. Independent variables of interest included measures of disorder, attractiveness, activity settings, and supporting assets. This study was approved by Tulane University IRB (15-794106U).

### 2.2. Data Collection Instruments and Procedures

Parks in two low-income, African American neighborhoods were assessed in spring and summer of 2016 and 2017. Neighborhood boundaries were defined by census tracts, and all parks within the neighborhood census tracts were included in the study. Using visual guides, pairs of trained observers conducted an environmental assessment of each park’s assets, attractiveness, condition, signs of disorder, and accessibility with a standardized, validated park audit tool, the Bedimo-Rung Assessment Tools–Direct Observation (BRAT–DO) (Louisiana State University, New Orleans, LA, USA) instrument. The tool has an overall reliability of 86.9% and validity of 78.7% [41]. Observers completed over six hours of classroom and field training and engaged in consensus building to establish consistent ratings on these measures. All observers were certified by a gold standard rater before data collection.

Using maps and aerial photographs of each park, pairs of observers walked the park and recorded information on park characteristics. Park activity settings included basketball courts, tennis courts, sports fields, playgrounds, greenspaces, pools, and walking paths. Supporting park assets included benches, bike racks, shelters, restrooms, drinking fountains, picnic tables, water features, and public art. Activity settings were recorded by the number of assets (i.e., 2 basketball courts), and supporting park assets were measured by presence or absence of the feature.

In measuring disorder, the presence of four items related to disorder (amount of litter present, amount of trash present, amount of visible “risky” litter, and visible graffiti) was recorded. Large items that would require an organized effort to remove, such as construction debris or piles of discarded items, were considered trash. Litter was assessed as small items that an individual could remove, such as cups or food wrappers. Items like condom wrappers, drug paraphernalia, or beer bottles were considered ‘risky’ litter. Signs of disorder were assessed on a scale of 1 to 5, with 1 being none present to 5 being a lot present. If any of the four items had a rating score of 3 or more, the park was categorized as having signs of disorder and coded 1. Those parks with ratings under 3 on all of these items were categorized as having little to no signs of disorder and coded 0. Overall, park attractiveness, which was based on landscaping and visual appeal, was evaluated on a scale of 1 (very unattractive) to 5 (very attractive). Attractiveness scores were dichotomized and coded into 0 for unattractive (ratings 1–2) and 1 for neutral/attractive (ratings 3–5).

To measure park use and park-based physical activity, the System for Observing Play and Recreation in Communities (SOPARC) tool, a validated tool, was used [42,43]. SOPARC applies momentary time sampling techniques to systematically collect information about individuals within predetermined park target areas. Using this tool, trained observers conducted periodic scans, a visual sweep from left to right, of target areas and recorded gender, age (youth versus adult), and activity level of each observed individual. Physical activity level was collected as sedentary (lying down, sitting, or standing in place), walking (casual pace), or vigorous (greater than an ordinary walk) [42]. For analytic purposes, moderate and vigorous physical activity were combined into one category labeled active. Scans were conducted six times per day (two morning, two noon, and two evening hours) over two weekdays and one weekend day. To capture park usage, optimum observation times were determined through consultation with neighborhood coalition members. Observations were summed to form park aggregates.

### 2.3. Statistical Analyses

Descriptive statistics of the park users, activity levels, park activity settings, and park conditions were calculated. To examine the relationship between park characteristics and park use, models were estimated in which number of total users, number of users by age (adult and youth), number of adults by gender (male and female), and number of users by gender by activity level (sedentary and active) were the dependent variables. Independent variables included signs of disorder, attractiveness, number of activity settings, and supporting assets. Park size and time of data collection were considered as confounders.

Count regression models were applied to the number of users in the parks. Tests of the model showed that Poisson regression was not appropriate as the dependent variables were over-dispersed, and not normally distributed. Negative binomial regression was applied to model park users, and examined the number of users across different conditions. Results from the models were reported as incidence rate ratios (IRRs), which denoted differences in the number of users and user activity level for a park with the variable of interest compared to a park without the variable.

## 3. Results

The 31 parks under study serve a population of 35,313 residents. The population density around these parks is an average of 4437 residents per square mile. Over 88% of the neighborhood residents are African American, and the median annual household income is $27,025. Comparatively, in New Orleans, 59% of the 382,922 residents are African American, and the median household income is $38,681 [44]. Park sizes ranged widely from 0.15 to 3200 acres (median = 2.81 acres). Parks varied from small, neighborhood parks to larger, linear parks that bordered waterways.

In the parks, the median number of activity settings was 4 (range: 1–9) and median number of supporting assets was 2 (range: 0–6). All park activity settings and supporting assets, except for pools and some restrooms, were accessible and open to all park users. On attractiveness, 19.4% of the parks were rated as unattractive (*n* = 6). Almost half of the parks (*n* = 15) exhibited signs of disorder. A moderate amount of litter was present in 14 (45.2%) of the parks, trash in 4 (12.9%) parks, risky litter in 4 (12.9%), and graffiti in 4 (12.9%). The disorder variable was a composite of these items. The descriptive statistics of park conditions and activity settings collected using the BRAT–DO are shown in Table 1.

Using SOPARC, a total of 278 observations were conducted, and 428 people were present within the 31 parks. Of the park users, 278 (65%) were observed being physical active (moderate to vigorous) and 150 (35%) were sedentary. Table 2 shows the distribution of park users. The majority observed were adults (*n* = 268, 63%) and were female (*n* = 250, 58.4%).

The results of the negative binomial models are summarized in Table 3 and Table 4. Disorder and park attractiveness were associated with park use across all users. Parks with signs of disorder were associated with 36% fewer park users (IRR = 0.64, 95% Confidence Interval (CI) = (0.45–0.91)). Parks rated as attractive were associated with 72% more park users (IRR = 1.72, 95% CI = (1.09–2.74)). Supporting assets and number of activity settings were not significant for all users.

When examining variables by population subgroups, results differed. For youth, attractiveness (IRR = 2.01, 95% CI = (1.08–3.74)) and number of activity settings (IRR = 1.00, 95% CI = (1.00–1.34)) were associated with park use. None of the variables were significant for males overall; whereas, disorder and attractiveness were significant for females overall. Among females, signs of disorder were associated with 49% fewer users (IRR = 0.51, 95% CI = (0.34–0.77)) and parks that were attractive were associated with 146% more users (IRR = 2.46, 95% CI = (1.39–4.33)).

The relationships between park conditions and park users varied by level of physical activity (Table 4). For active male and sedentary female park users, there were no relationships between number of users and disorder, park attractiveness, number of activity settings, or supporting assets. Signs of disorder were associated with 45% fewer sedentary male users (IRR = 0.55, 95% CI = (0.32–0.80)). Disorder was associated with 52% fewer active female users (IRR = 0.48, 95% CI = (0.31–0.73)). Active female use was also positively associated with park attractiveness (IRR = 2.38, 95% CI = (1.27–4.48)).

## 4. Discussion

Using direct observations, this study examined whether signs of disorder, attractiveness, activity settings, and supporting assets were associated with park visitation and levels of physical activity in low-income, African American neighborhoods. Results varied by gender and user activity level. Park attractiveness and disorder were associated with both park usage and park-based physical activity among females. Females, specifically active females, were more likely to be present in attractive parks and parks with fewer signs of disorder. For youth, number of activity settings and park attractiveness were associated with park use.

Several studies have found results that support these associations. Specific park characteristics, such as incivilities, condition of assets, and quality of activity settings, have been associated with number of users and physical activity levels [27,33,34,35]. Previous research has shown that community residents report vandalism, trash, graffiti, and litter as barriers to park use and physical activity [31,32,35,45]. Signs of disorder and/or deferred maintenance may deter park use due to concerns about safety [31,32,45]. In interviews and surveys, park aspects that are related to attractiveness (overall aesthetics, flowers, trees, gardens, bushes, scenery, bird life, and water features) were reported as important for park use, and motivators for physical activity [31,32,45,46,47,48]. Study participants have also stated that pleasant sights, sounds, and smells impacted park use and activity [46,49]. Improved landscaping, installation of visual elements, and remediating disorder in parks and park activity settings may facilitate park usage. Specifically among minority women, higher-quality, well-maintained parks with fewer signs of disorder have been found to be an important determinant of physical activity [50].

While these results did not find an association between park use and activity settings overall, there was an association between park use and activity settings for youth. Activity settings have been found to increase park and playground use among youth [51,52,53]. In one study, greater number of park amenities was associated with greater odds of vigorous physical activity among youth [54]. The improvement of parks through the addition of activity settings may have the ability to increase park use and physical activity among youth within low-income, African American communities.

Research has shown that improvements to parks can result in more usage and more physical activity [55]. Our quantitative findings, along with previous research, suggest that city governments and neighborhood leaders may consider increasing park maintenance and addressing attractiveness in parks as relatively low-cost strategies to potentially encourage park use and physical activity among women in low-income, African American neighborhoods.

## 5. Strengths and Limitations

One of the strengths of this study is the use of two validated, observational instruments for the collection of data on both park conditions and actual park users. Another strength is the setting. The neighborhoods in this study are primarily African American, and facilitators and barriers to park use are not well-studied in this setting. Additionally, all parks in the neighborhoods were included in the study, and the activity observed may be representative of recreational activity. Future research might include combining direct observations with interviews or self-reports to understand better park user perceptions and factors associated with physical activity.

This study has several limitations. First, park user observations occurred at fixed moments and may not be reflective of general patterns of park use. While observations were conducted on different days of the week and times of day to capture usage, data were not collected during different seasons and weather. Second, although observers were trained and certified, some of the measured constructs are subjective, and observers may introduce bias. This may be especially true in measuring attractiveness as this feature may be more difficult to capture than other more objective features. Third, confounding factors that were not measured, and may impact results may include park design factors (i.e., short sight lines or hidden areas), park distance to other community resources (i.e., stores, churches, schools, or libraries), population density surrounding the park, crime, signs of social disorder, and neighborhood walkability. These factors could be the focus of future studies. In addition, these parks are within low-income, African American neighborhoods in an urban setting in Louisiana, and the results may not be generalizable to other settings.

## 6. Conclusions

As parks can be a resource for neighborhood-based physical activity, understanding and addressing factors that may influence park use can be important for enhancing physical activity opportunities [4,24,25,26,27,28,29]. While further research is needed for a better understanding of the relationship between park conditions, park usage, and physical activity, this study contributes to the evidence that park disorder and attractiveness may impact park use, specifically among women in low-income, African American communities. While residents may have access to parks, the conditions of the parks may limit or deter use. By making investments in clean-up efforts and improving park resources that already exist to create more attractive and inviting settings, communities may be able to facilitate more park usage, increased park-based physical activity, and improved health. These data provide support for this type of low-cost environmental change to improve the built environment.

## Figures and Tables

**Table 1:** Descriptive statistics for park characteristics (*n* = 31), New Orleans, Louisiana.

Park Characteristic	Number of Parks (%)
Disorder	15 (48%)
Attractive	25 (80%)
*Activity settings*	
Basketball courts	17 (55%)
Tennis courts	3 (10%)
Sports fields	18 (58%)
Playgrounds	19 (61%)
Greenspaces	29 (94%)
Pools	3 (10%)
Walking paths	19 (61%)

**Table 2:** Observed park user characteristics (*n* = 428), New Orleans, Louisiana.

Characteristics	*n*	Age *		Activity Level	
		Youth *n* (%)	Adult *n* (%)	Sedentary *n* (%)	Active *n* (%)
Total park users observed	428	149 (37%)	268 (63%)	150 (35%)	278 (65%)
Male	178	57 (32%)	120 (67%)	85 (48%)	93 (52%)
Female	250	92 (37%)	148 (59%)	65 (26%)	185 (74%)

* Age was not recorded for 11 individuals.

**Table 3:** Negative binomial regression models for relationships between park attributes and park users by age and gender, (*n* = 278 observations), New Orleans, Louisiana.

Park Attributes	Park Users									
	All Users *n* = 428		Youth *n* = 149		Adults *n* = 268		Males *n* = 178		Females *n* = 250	
	IRR	95% CI	IRR	95% CI	IRR	95% CI	IRR	95% CI	IRR	95% CI
Signs of Disorder	0.64	(0.45–0.91) **	0.95	(0.60–1.52)	0.56	(0.38–0.81) **	0.92	(0.60–1.40)	0.51	(0.34-.77) **
Attractive	1.72	(1.09–2.74) *	2.01	(1.08–3.74) *	1.47	(0.89–2.46)	1.09	(0.63–1.90)	2.46	(1.39–4.33) **
Activity settings	1.09	(0.97–1.23)	1.15	(1.00–1.34) *	1.04	(0.92–1.17)	1.02	(0.89–1.16)	1.10	(0.97–1.25)
Support Assets	1.04	(0.89–1.22)	0.95	(0.78–1.16}	1.09	(0.93–1.30)	1.15	(0.96–1.38)	1.00	(0.84–1.19)

Reported in Incidence Rate Ratios (IRR), 95% Confidence Interval (CI). * *p* <0.05, ** *p* < 0.01.

**Table 4:** Negative binomial regression models for relationships between park attributes and park users by level of physical activity and gender, (*n* = 278 observations), New Orleans, Louisiana.

Park Attributes	Park Users							
	Active Males *n* = 93		Sedentary Males *n* = 85		Active Females *n* = 185		Sedentary Females *n* = 65	
	IRR	95% CI	IRR	95% CI	IRR	95% CI	IRR	95% CI
Signs of Disorder	1.63	(0.96–2.75)	0.55	(0.32–0.94) *	0.48	(0.31–0.73) **	1.11	(0.61–2.01)
Attractive	1.46	(0.71–2.98)	0.88	(0.45–1.74)	2.38	(1.27–4.48) **	2.27	(0.97–5.31)
Activity settings	1.04	(0.89–1.22)	0.98	(0.84–1.14)	1.08	(0.95–1.23)	1.10	(0.93–1.32)
Support assets	1.21	(0.99–1.50)	1.06	(0.84–1.32)	1.17	(0.98–1.40)	0.78	(0.60–1.02)

Reported in Incidence Rate Ratios (IRR), 95% Confidence Interval (CI). * *p* < 0.05, ** *p* < 0.01.
